# Supplementary material for: A Structural Potential of Rare Trinucleotide Repeat Tracts in RNA
Source: Int J Mol Sci. 2022 May 23;23(10):5850. doi: 10.3390/ijms23105850 (PMC9144543; doi:10.3390/ijms23105850)
Supplement: Supplementary file 1 [file ijms-23-05850-s001.zip › Figure S1.pdf]

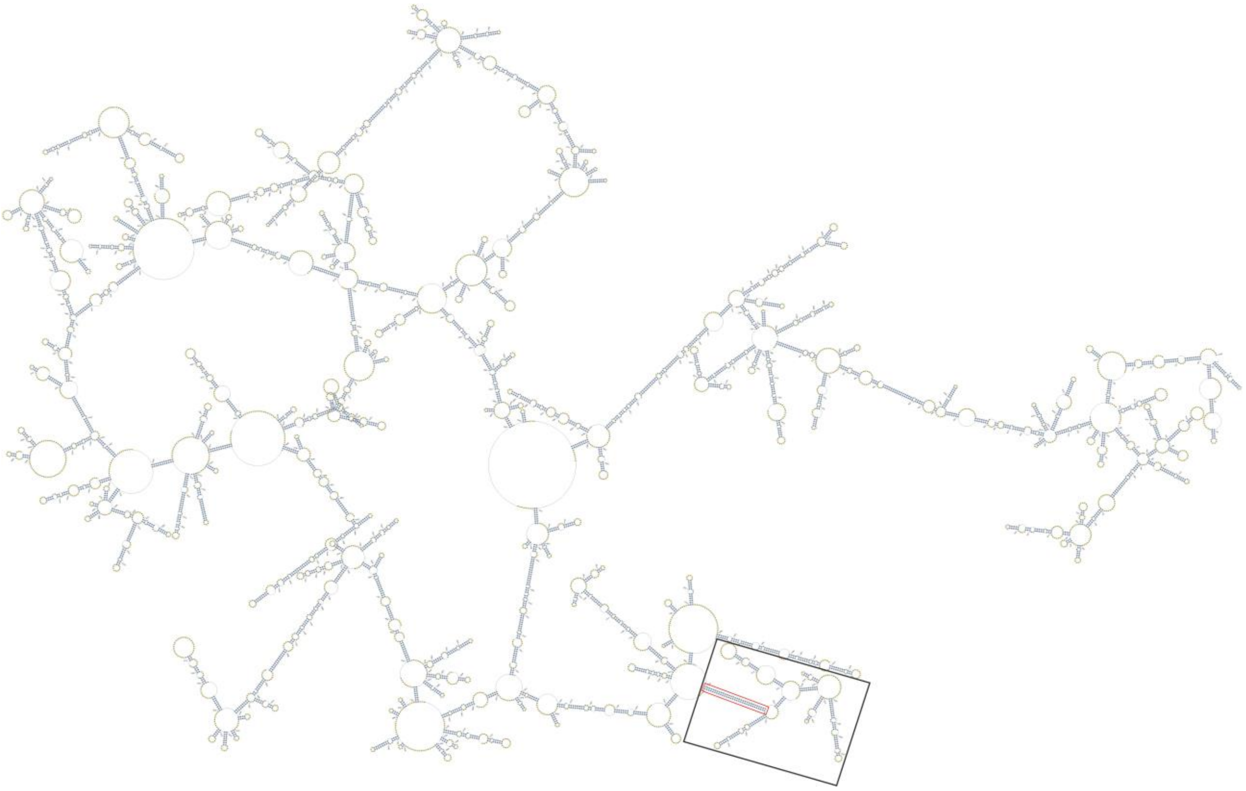

Figure S1. In silico secondary structure of GABRA4 whole 3'UTR (RNAFold). Analyzed region is marked in black frame, interacting repeat tracts are marked in red frame.
