# Supplementary material for: A Structural Potential of Rare Trinucleotide Repeat Tracts in RNA
Source: Int J Mol Sci. 2022 May 23;23(10):5850. doi: 10.3390/ijms23105850 (PMC9144543; doi:10.3390/ijms23105850)
Supplement: Supplementary file 1 [file ijms-23-05850-s001.zip › Figure S2.pdf]

A

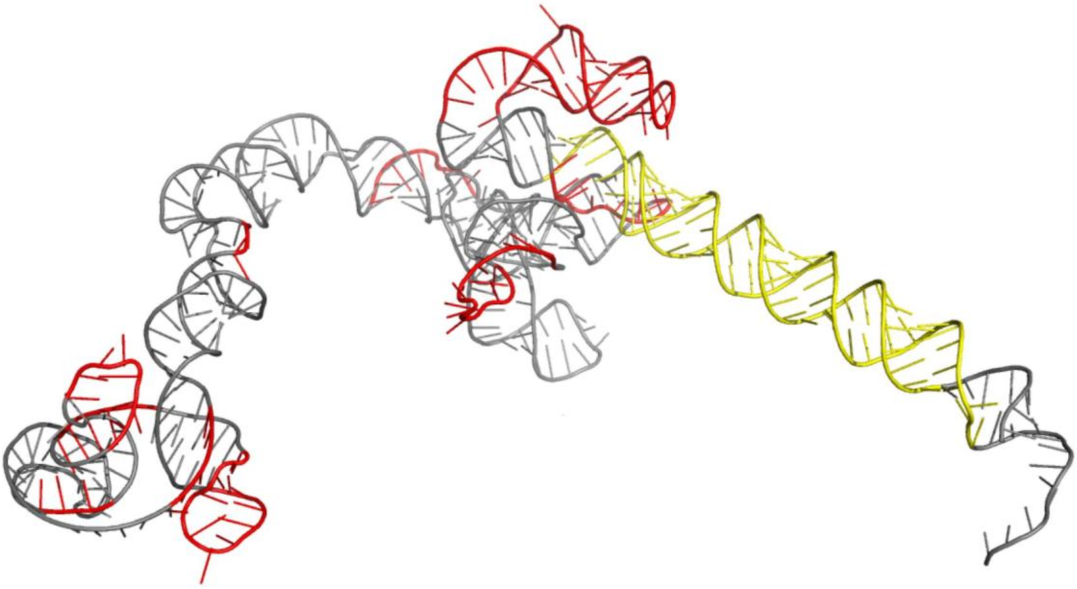

B

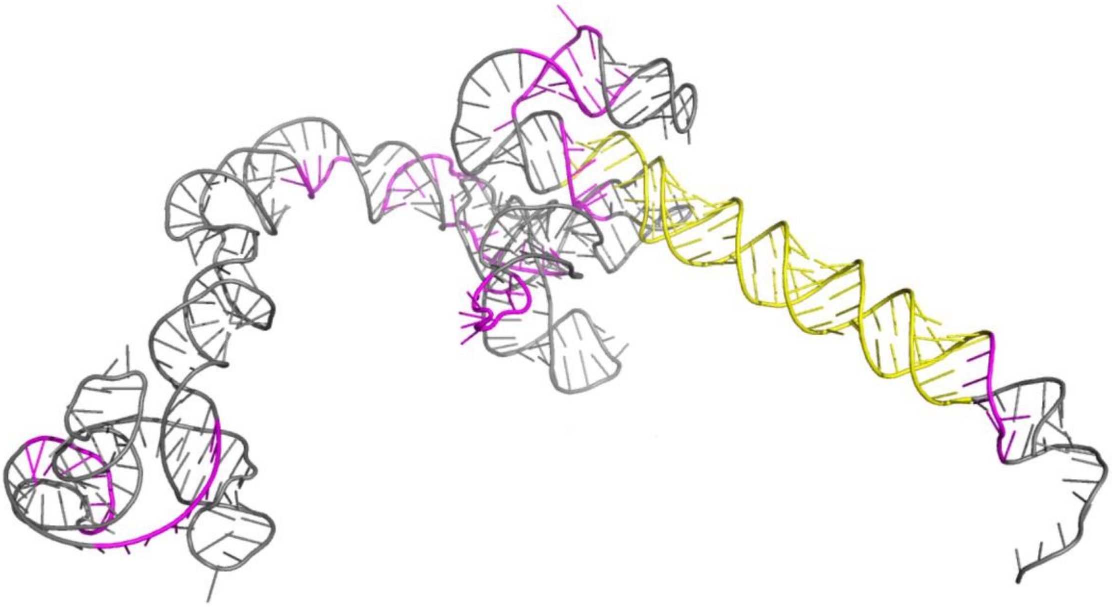

Figure S2. GABRA4 3D structure with reactive (red) sites in the chemical mapping (A) and miRNA target sites (magenta) prediction (B). AAU-GUU repeats are marked yellow.
