# Supplementary material for: A Structural Potential of Rare Trinucleotide Repeat Tracts in RNA
Source: Int J Mol Sci. 2022 May 23;23(10):5850. doi: 10.3390/ijms23105850 (PMC9144543; doi:10.3390/ijms23105850)
Supplement: Supplementary file 1 [file ijms-23-05850-s001.zip › Figure S3.pdf]

Target Rank	Target Score	miRNA Name	Sequence (bold=seed)	Gene Symbol	Seed Location within target	site (3'UTR/analyzed domain)	miR-Expression	TSI	evidence
6	100	hsa-miR-126-5p	C**AUUAUUA**CUUUUGGUACGCG	GABRA4	**2455, 2467, 2470, 2473, 2476, 2479, 2482, 2485, 2488, 2491, 2494, 2497, 2500, 2503**, 2701, 2703, 4015, 4474, 4856	AAU repeats	spleen, thyroid	0,6-0,84	experimental, cloned
4	100	hsa-miR-4795-3p	A**UAUUAUU**AGCCACUUCUGGAU	GABRA4	**2454, 2466, 2468, 2471, 2474, 2477, 2480, 2483, 2486, 2489, 2492, 2495, 2498, 2501, 2504**, 2701, 4016, 4474, 4856	AAU repeats	no data (TA)	-	experimental
2	100	hsa-miR-5692a	C**AAAUAAU**ACCACAGUGGGUGU	GABRA4	195, 693, 869, 1961, **2204, 2432**, 2571, 7936, 8908	2204/94, 2432/322	brain	0,33-0,48	experimental
11	99	hsa-miR-607	G**UUCAAAU**CCAGAUCUAUAAC	GABRA4	307, **2383**, 3063, 6261, 7291, 8384, 8932	2383/273	no data (TA)		experimental
20	98	hsa-miR-6507-5p	G**AAGAAU**AGGAGGGACUUUGU	GABRA4	78, **2369**, 3596, 3618, 4314, 5391, 8689	2369/259	stomach, testis, kidney	0,62-0,88	experimental
28	97	hsa-miR-3671	A**UCAAAU**AAGGACUAGUCUGCA	GABRA4	306, **2383**, 7290	2383/273	no data (TA)		experimental
50	93	hsa-miR-3171	A**GAUGUAU**GGAAUCUGUAUAUAU	GABRA4	**2323**, 2601, 7728	2323/213	muscle, bone, adipocyte, brain	0,74-0,76	experimental
56	92	hsa-miR-205-5p	U**CCUUCAU**UCCACCGGAGUCUG	GABRA4	859, **2309**, 4113, 4421, 8425	2309/199	skin	0,94-1	experimental, cloned
80	86	hsa-miR-33a-3p	C**AAUGUUU**CCACAGUGCAUCAC	GABRA4	478, **2412**, 9257	2412/302	no data (TA)	-	experimental, cloned
87	85	hsa-miR-5007-3p	AUCAUAUGAACCAAACUCUAAU	GABRA4	**2306**, 7674, 8533	2306/196	no data (TA)	-	not experimental
89	84	hsa-miR-196a-1-3p	CAACAACAUUAAACCACCCGA	GABRA4	**2120, 2123, 2126, 2129, 2132, 2135, 2138, 2141, 2144, 2511**, 7899	GUU repeats	no data (TA)	-	not experimental
97	84	hsa-miR-5571-5p	C**AAUUCU**CAAAGGAGCCUCCC	GABRA4	1458, **2355**, 7275, 9116	2355/245	testis, gallbladder, bone marrow	0,37-0,5	experimental
142	81	hsa-miR-3529-3p	AACAACAAAAUCACUAGUCUUCCA	GABRA4	**2119, 2122, 2125, 2128, 2131, 2134, 2137, 2140, 2143**, 8515	GUU repeats	no data (TA)	-	not experimental
139	81	hsa-miR-676-5p	U**CUUC**AACCUCAGGACUUGCA	GABRA4	1221, **2385**, 4111, 4468	2385/275	no data (TA)		experimental

Figure S3. miRDB top search results found for *GABRA4* 3′UTR region. TA – according to miRNA Tissue Atlas [56].
